# Even the COVID-19 pandemic didn´t change anything: insights from a trend study on the cooperation of general practitioners and occupational health physicians in Germany

**DOI:** 10.1186/s12875-026-03463-7

**Published:** 2026-07-09

**Authors:** Monika A. Rieger, Anke Wagner, Martina Michaelis, Stefanie Joos, Stefan Wilm

**Affiliations:** 1 Institute of Occupational and Social Medicine and Health Services Research, Faculty of Medicine of the University Tübingen, University Hospital Tübingen, Wilhelmstraße 27, Tübingen, 72074 Germany; 2 Freiburg Research Centre for Occupational and Social Medicine, Bertoldstraße 63, Freiburg, 79098 Germany; 3 Institute for General Practice and Interprofessional Care, Faculty of Medicine of the University Tübingen, University Hospital Tübingen, Osianderstraße 5, Tübingen, 72076 Germany; 4 Institute of General Practice, Centre for Health and Society, University Hospital Düsseldorf, Moorenstraße 5, Düsseldorf, 40225 Germany

**Keywords:** General Practitioners, Occupational Health Physicians, Humans, COVID-19, Return to Work, Prejudice, Counselling, Surveys and Questionnaires, Regression Analysis, Retrospective Studies

## Abstract

**Background:**

The working fields of general practitioners (GPs) and occupational health physicians (OHPs) overlap manifoldly. Yet, cooperation between both groups is often scarce as has been revealed by studies from several countries. During the COVID-19 pandemic, GPs dealt with many work-related counselling topics. We conducted a trend study surveying German GPs and OHPs in 2014/2015 (GPOP-0 study) and 2023/2024 (GPOP-Trend) to analyse trends regarding a potential change of attitudes towards interdisciplinary cooperation over time, before and after the COVID-19 pandemic.

**Methods:**

The trend study used repeated cross-sectional surveys and an identical questionnaire for GPs and OHPs, which was sent by postal mail to both groups. The questionnaire covered topics regarding cooperation and attitudes towards both professional groups, as well as questions to assess the perceived quality of cooperation with regard to counselling purposes specific for the COVID-19 pandemic (only in GPOP-Trend). The statistical analysis followed a prior published analysis plan, and comprised an exploratory factor analysis, Mann-Whitney U tests, and regression analyses.

**Results:**

More than 1,000 physicians took part in both surveys (GPOP-0: 585 GPs and 473 OHPs, response rate 35%; GPOP-Trend: 482 GPs and 532 OHPs, response rate: 30%). We identified hardly any cooperation between these two groups during the COVID-19 pandemic. In cases where cooperation took place, it was rated more positively by OHPs than by GPs in retrospect. Comparing the total survey data of both time points revealed that attitudes among GPs and OHPs changed only little over time. The strongest predictor for the attitudes surveyed was the variable “professional group” in all regression models. This predictor was also the most relevant, when investigating the influence of different variables during the COVID-19 pandemic on attitudes towards cooperation of GPs and OHPs.

**Conclusions:**

Socialization within a professional group seems to be the determining factor, and therefore attitudes remain stable as described by Social Identity Theory. We assume that interventions that focus solely on strengthening cooperation between GPs and OHPs therefore have only a very limited effect. We therefore suggest another approach with interventions targeting primarily on patient outcomes and the design of clear interfaces or clinical pathways.

**Supplementary Information:**

The online version contains supplementary material available at 10.1186/s12875-026-03463-7.

## Background

General practitioners (GPs) and occupational health physicians (OHPs) play a key role, especially in challenging times for healthcare systems due for example to rising shortage of physicians [1] and consequences of demographic change [2], and have many overlapping working fields (e.g. general health, support of chronically ill people in working age, prevention, rehabilitation, and return to work processes). German OHPs focus on the interaction between work and health but are for example not involved in the medical treatment of patients [3]. In most cases they are employed by companies, and responsible for specific work-related and general health problems of employees [3]. OHPs focus on prevention activities such as structural (e.g. improving working conditions) and behavioral prevention at workplaces and individual preventive measures (e.g., vaccinations, preventive medical check-ups), and are also available for medical consultations with employers and employees [3].

Against the background of longer working life and work-related hazards derived from the changing world of work [4], the cooperation of the two professional groups GPs and OHPs is becoming more and more important, and should be promoted to improve patient care [5]. Research on cooperation between professional groups is an important part of health services research and helps to further develop the healthcare system [6]. The work of OHPs is specifically addressed in work-related health services research [7] or occupational health services research [8]. So far, research focusing on cooperation of GPs and OHPs has been mainly conducted in Central Europe [3, 5, 9–14]. One reason for the limited research activities might be that in some countries other health professionals instead of physicians engage in occupational health, e.g. occupational health nurses or occupational hygienists [15–17], or that in many countries occupational health services are neither legally required nor encouraged by the government or society [18].

In Germany a comprehensive research project has been carried out by our research group encompassing a systematic literature review to identify the current status in literature regarding the cooperation between GPs and OHPs in 2011 [14], and a qualitative study with focus group interviews to explore deficits, barriers, and possible areas for improvement in 2009 [13, 19]. Based on the literature review and the qualitative study, a standardized questionnaire was developed [20] and a written postal survey among GPs and OHPs performed in 2014/2015 in the federal states of Baden-Württemberg and North Rhine-Westphalia [3]. A subgroup analysis within the cross-sectional study revealed that the participants in the federal state of Baden-Württemberg, Germany (313 GPs and 184 OHPs) considered their responsibilities in the working field as clearly separated, but also showed interest in improving the cooperation [3].

To sum up, so far, mostly studies with cross-sectional design have been conducted. Studies with a longitudinal research design are currently missing, and can identify further important aspects for cooperation between these two groups. This approach might be particularly fruitful when events with a potential impact on the research topic occurred during the observation period [21], as was the case with the COVID-19 pandemic.

During the COVID-19 pandemic, GPs and OHPs were challenged in many ways. With regard to people of working age, besides implementation of public health measures, care for and counselling of individuals with increased COVID-19 risk was an essential task for both professional groups. Advising patients / employees or employers whether or how employees could work on site without being exposed to an increased risk of infection required detailed knowledge of the individual´s health conditions together with the specificity of the workplace. In this context, it was helpful for the OHPs and employees if there was direct communication between the GPs and the OHPs, subject to medical confidentiality. Not least in view of the prolonged duration of the pandemic and the associated high risk of infection in public spaces or on site at the workplace, it was essential that employees at increased risk were also able to work, and that their work participation was ensured rather than being placed on sick leave. Return to work after a severe course of COVID-19 or for patients suffering from Long COVID or Post COVID is another challenging and continuing task for GPs and OHPs and benefits from a careful clinical and occupational health approach, too [22].

As SARS-CoV-2 infection has been prevalent among people of working age and the workplace setting has been a major setting for individual prevention, prevention regarding individuals with increased risk of infection or a severe course of the disease as well as public health measures to protect against the spread of the infection, we hypothesized that GPs and OHPs collaborated naturally more during the years of the COVID-19 pandemic and may collaborate with regard to rehabilitation and return-to-work of patients suffering from Long- or Post-COVID syndrome afterwards. We therefore regard the COVID-19 pandemic as a “natural experiment” in which we can observe the cooperation experiences of GPs and OHPs. Natural experiments occur naturally and comprise different circumstances which cannot be controlled by researchers in contrast to randomized-controlled trials [23]. The COVID-19 pandemic served as a natural experiment as it had a significant impact on infrastructure, policies, services, and especially healthcare systems [23]. Therefore, we hypothesized that this intensified experienced cooperation during the COVID-19 pandemic between OHPs and GPs could be associated with a change in attitudes regarding cooperation and shared fields of action, which we address in our study.

We haven´t found any study examining the cooperation between GPs and OHPs regarding the COVID-19 pandemic. In order to overcome this situation, we performed a written postal survey in 2023/2024 after the COVID-19 pandemic addressing GPs and OHPs in Germany, and – by repeating the written postal survey performed in 2014/2015 [3] – were able to conduct a trend study.

The following paper presents the results of this trend study. In particular, we focus on the following research questions (RQ):

- RQ 1: How was cooperation between GPs and OHPs experienced by them during the COVID-19 pandemic in retrospect?
- RQ 2: What differences can be found in the attitudes towards cooperation regarding the respective other professional group, and are there any changes in the attitudes of GPs and OHPs towards cooperation over time?
- RQ 3: Which sociodemographic and job-specific characteristics and individual experiences with cooperation during the COVID-19 pandemic have an influence on attitudes towards cooperation?
- RQ 4: Which sociodemographic and job-specific characteristics have an influence on attitudes of GPs and OHPs towards cooperation over time (2014–2024)?

## Methods

### Study design

Based on our previous work [3], we performed a trend study using cross-sectional data from two repeated surveys in 2014/2015 and 2023/2024 to answer our research questions. “A trend study is the analysis and description of a trend and its impact on a selected area (…).” [24] We consider this design well suited for representing the cooperation experiences of these two groups over time. By reporting this study, we followed the guidelines of the STROBE statement [25].

### Study sample characteristics, setting and data collection

The data collection in the GPOP-0 study is described in detail by Moßhammer et al. [3]. We aimed to question a sample size of at least 1,000 GPs and OHPs, and followed thereby some of the recommendations of Edwards et al. to increase the response rates of the questionnaire (e.g., using personalised materials and stamped return envelopes, assuring confidentiality, including intensive follow-up and originating from a university) [26].

A written postal survey of GPs and OHPs in 2014 and 2015 was conducted to assess and compare the experiences and attitudes regarding cooperation with the respective other professional group by means of a cross-sectional study [3], and a second independent cross-sectional study surveying GPs and OHPs in 2023 and 2024 in the same geographical regions of Germany (federal states). In the following, we refer to the first survey in 2014/2015 as GPOP-0 study and to the recent survey in 2023/2024 as GPOP-Trend.

The questionnaire in the GPOP-0 study – as well as a reminder after four weeks – was sent by mail.

- to a random sample from 5,430 freely accessible addresses of the Association of Statutory Health Insurance Physicians with 1,000 GPs respectively in two large German federal states: Baden-Württemberg (BW, in the year 2014, www.kvbawue.de) and North Rhine-Westphalia (NRW, in the year 2015, www.kvno.de, www.kvwl.de) who received the postal mailing by the respective regional academic institution for general practice (University of Tübingen, University of Düsseldorf), and.
- to all 1,062 members of the Association of German Business and Company Doctors (VDBW, Verband Deutscher Betriebs- und Werksärzte e. V.) aged ≤ 65 years in the year 2014 from BW and NRW as well as three additional federal states (Schleswig-Holstein, Brandenburg, Lower Saxony) and one city state (Hamburg) to reach the sample size of *n* = 1,000. The questionnaire and study information together with an invitation letter by the academic institution for occupational medicine (University of Tübingen) was sent by the VDBW itself with a recommendatory letter because of anonymity reasons.

For the GPOP-Trend study in 2023/2024, we used the same methodical approach with only few modifications. Again, we performed a postal data collection and used a postal reminder after four weeks to reach at least about 1,000 GPs and OHPs. The questionnaire was sent by mail.

- again, to the random sample from freely accessible addresses of the Association of Statutory Health Insurance Physicians with 1,100 GPs in BW (in the year 2023) and 1,000 GPs in NRW (also in the year 2023).
- to all 1,297 members of the VDBW from BW and NRW at the beginning of the year 2024. Since there were more VDBW members in BW and NRW than in the year 2014, no further federal states were required to achieve the targeted sample size of *n* = 1,000. In the GPOP-Trend study no age limit was set for OHPs. Similar to the procedure in 2014, the questionnaire was sent by the VDBW itself with an accompanying recommendations letter because of anonymity reasons.

Both cross-sectional studies (GPOP-0 and GPOP-Trend) were approved by the Ethics Committee of Medical Faculty and University Hospital of the University of Tübingen (Project-No. for GPOP-0 study: 558/2013BO2; Project-No. for GPOP-Trend: 534/2023BO2). By developing the study protocol of the GPOP-Trend, we collaborated with the data protection department to correctly fulfil the requirements of the General Data Protection Regulation of the European Union (GDPR) and the respective German regulation. The participants were informed in detail about the study and gave their written consent by sending back the completed questionnaire in the stamped return envelopes.

### Questionnaire

At both time points, we utilized an almost identical questionnaire for GPs and OHPs. This questionnaire is described in detail in Moßhammer et al. [3]. The standardized questionnaire is based on a systematic literature research [14] and three qualitative focus group interviews with GPs, OHPs and physicians working on both fields [13, 19, 20]. For the analysis presented here, we refer to the following topics in the questionnaire:

- Sociodemographic and job-specific characteristics included the variables age, gender, years of professional experience, years in the medical specialty (i.e. working as GP or OHP), medical specialty and additional qualification.
- Experiences with cooperation during the COVID-19 pandemic: Only in the GPOP-Trend survey, six new items were developed in a multidisciplinary working group to assess the perceived quality of cooperation of both professional groups with regard to counselling purposes specific for the COVID-19 pandemic (Likert scale of “1=agree not at all” to “5=agree definitely”): three items addressed the medical counselling of an individual with increased COVID-19 risk (experienced helpfulness of the interdisciplinary cooperation regarding own counselling or decisions for own measures, perceived quality of the counselling of the patient by the respective other profession), three similar items were added related to caring for individuals with Long COVID. Despite the clear definitions for Long COVID (longer-term health impairments after infection up to four weeks) and Post COVID (longer-term health impairments at least twelve weeks and longer) as for example outlined in the German medical guideline for patients with Long / Post-COVID [22], we intentionally used only the colloquial term ‘Long COVID’ [27] in the questionnaire, as we assumed that our respondents did not explicitly distinguish between Long and Post COVID. There was also an additional answer option for those who had not experienced these specific situations (see for further information Additional File 1).
- Attitudes towards cooperation of both professional groups assessed by 17 statements regarding common and separate working fields and responsibilities as well as professional self-perceptions/external perceptions (Likert scale of “1=agree not at all” to “5=agree definitely”).

In the GPOP-0 study, a pretest was conducted with six GPs and eleven OHPs. The pretest was repeated again for GPOP-Trend with two GPs and eight OHPs. There were only minor adjustments made to the GPOP-Trend questionnaire, such as the option to select a third gender.

### Data analysis

Every step of the data analysis is described in detail in the prior published statistical data analysis plan [28]. Data analysis was performed with IBM SPSS Version 29. Prior to the data analysis, the data entry of each questionnaire was carefully monitored, and quality assurance procedures were applied. The person monitoring the data was not involved in the data entry and controlled for consistency, quality, and completeness [29]. City size (number of inhabitants) was additionally determined by an internet search using the pseudonymized postal code, which was delivered by the VDBW and by the Association of Statutory Health Insurance Physicians.

This paper focuses on the following results: the experiences with cooperation during the COVID-19 pandemic (RQ 1) and the attitudes towards cooperation (RQ 2, RQ 3, RQ 4), i.e. statements regarding common and separate working fields and responsibilities as well as professional self-perceptions and external perceptions.

The data analysis for this paper therefore comprised further the following steps (for details, see [28]):

- Descriptive results of score values and single item values on the base of valid answers were presented by means of means, standard deviations, median, range and, if necessary, percentages for professional groups and survey time point separately. All descriptive anal-yses were derived from raw data. For further analyses, missing values were imputed by multiple imputation method in SPSS with five iterations [30].
- Experiences with cooperation during the COVID-19 pandemic: Development of sum scores depicting experiences with the cooperation with the respective other professional group when counselling an individual during the COVID-19 pandemic (only GPOP-Trend data): we constructed two sum scores from each of the respective three COVID-19-items considering individuals a) at increased COVID-19 risk and b) suffering from Long COVID. The un-standardized sum scores have a possible total range from 1 to 15 points. By reaching at least eight points, we rated the respective experiences during the COVID-19 pandemic as “positive”. We then computed sum scores differences between both professional groups by Mann-Whitney U tests. A p-value<0.05 was considered as statistically significant. We calculated the effect size with the formula r= z (Mann-Whitney U test)/root (N (total)) and categorized it with r < 0.10=small effect/difference, r < 0.50=medium effect/difference and r ≥ 0.50=large effect/difference [31]. The two sum scores served amongst other variables as possible predictors in further multivariate linear regression analyses of attitudes towards cooperation (see below).
- Attitudes towards cooperation: The 17 statements regarding common and separate working fields and responsibilities, and professional self-perceptions and external perceptions were analysed with regard to psychometric properties (structural validity by exploratory factor analysis, corrected item-to-total correlation (CITC) and by reliability analysis (Cronbach’s alpha) as well as floor and ceilings effects). Conditions of the factor analysis were: principal component analysis (PCA), eigenvalue criterion >1, Varimax rotation, Kaiser-Meyer-Olkin (KMO)-criteria test values for sampling adequacy of at least 0.5 and a significant Bartlett sphericity test for homogeneity of variances as appropriate condition for this approach. Items were regarded as good for factor loading on its own factor if >0.5 and on other factors if <0.3 [32]. Based on the total data set, the factor analysis revealed four factors depicting different attitudes towards cooperation of both professional groups (for more specific results of the exploratory factor analysis see Additional Files 2-4): factor 1 gathers items focusing on the work of OHPs and can be described by the saying “He who pays the piper calls the tune”, factor 2 addresses an critical attitude towards the work of GPs which we named “Well meant, but not done well”, factor 3 describes the attitude focusing on patients´ outcomes “Benefits for patient care through the involvement of occupational health physicians”, and factor 4 the rather critical attitude towards OHPs “Poaching in foreign hunting grounds”. These four factors were derived from the analysis of 13 out of 17 items and represented a stable solution across both survey time points. Thus, the single rather pointedly formulated statements reflecting deeply held positive or negative views towards the other professional group could be presented as easily describable attitudes (see Table 1).
  **Table 1:**
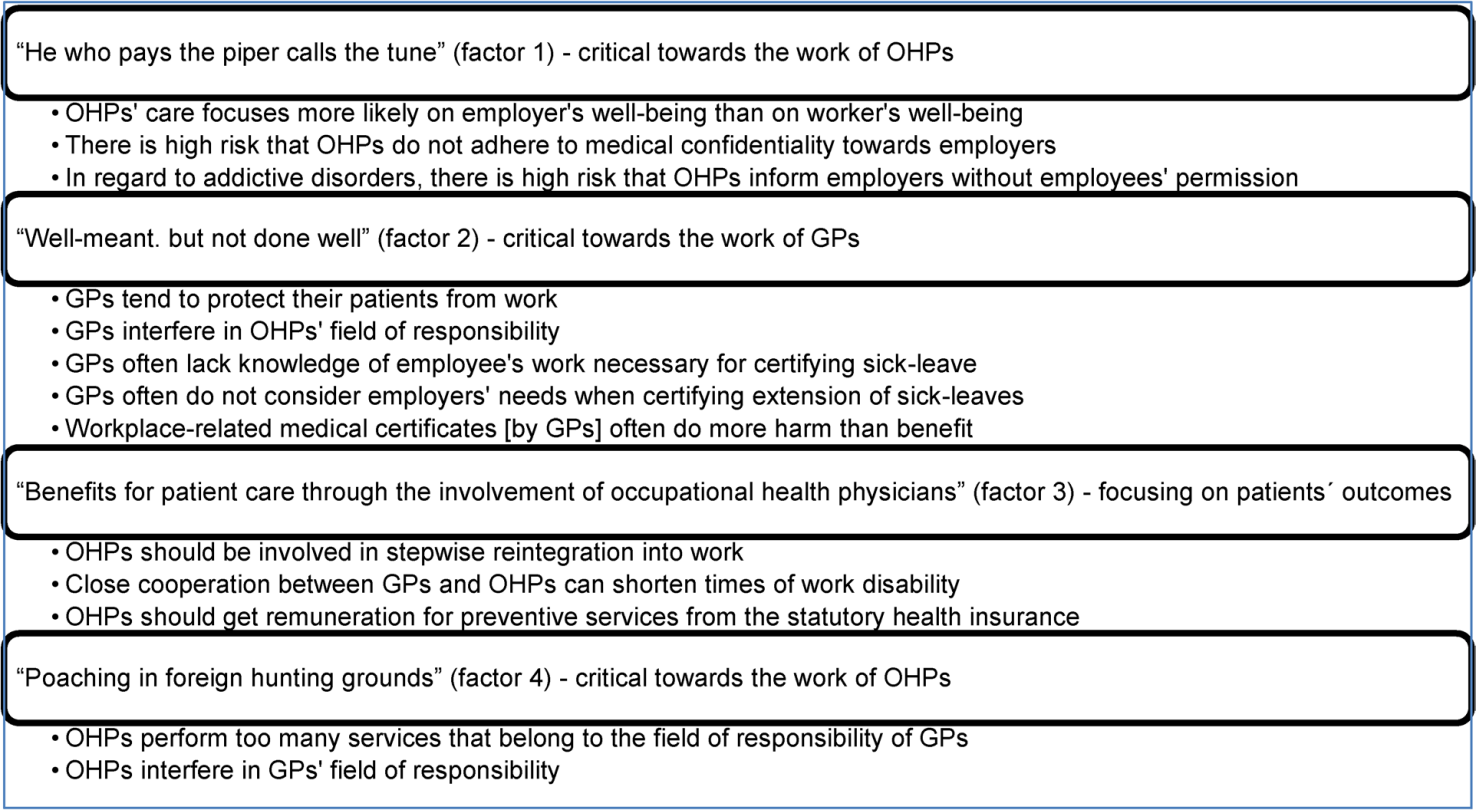
The four attitudes (factors) resulting from exploratory factor analysis and the respective single items depicting pointedly formulated views towards common and separate working fields and responsibilities

Stable result of the exploratory factor analysis across both survey time points; 13 out of 17 items included; excluded items: (4) GPs see OHPs as competition; (7) GPs feel criticised by OHPs when OHPs communicate remarkable medical findings to GPs, (6) GPs find OHPs' work helpful, (8) OHPs find GPs' work helpful. For more specific results of the exploratory factor analysis see Additional Files 2-4
- On the base of these four factors, descriptive statistics were conducted to describe the different attitudes. We also computed differences pro factor and differentiated between the two professional groups by Mann-Whitney U test. A p-value<0.05 was considered as statistically significant. We calculated and categorized the effect size according to Cohen’s suggestions (see above [31]). For each of the four attitudes derived from the factor analysis, a multivariate linear regression analysis using all data from both surveys GPOP-0 and GPOP-Trend was performed. The backward method was used to eliminate variables (*p*_*(out)*_=0.051) to reach a parsimonious model. Years in medical specialty served as adjusted predictor in the regression models, and we adjusted for time point of the survey and professional group. The other sociodemographic and job-specific characteristics (age, gender, years of professional experience) were included in the models as possible mediators. Further, we added a double qualification as GP and OHP to the analysis, as about 4% of all responding GPs were qualified additionally in occupational medicine and also 38% of all OHPs in the sample were qualified as specialist for general or internal medicine (see Table 2). Requirements such as tests for normal distribution and exclusion of multicollinearity were checked. The latter is defined as very strong correlation between predictor variables (variance inflation factor>10 and/or tolerance value<0.2 and even better <0.1 [33]).

## Results

### Response rates and sample characteristics

In the GPOP-0 study, questionnaires from 585 GPs and 473 OHPs were available (response rate: 35%). In GPOP-Trend, 482 GPs and 532 OHPs participated in the survey (response rate: 30%). Separated by professional group, the overall response rate was lower in GPs (26%; 29% in GPOP-0, 23% in GPOP-Trend) than OHPs (43%; 45% in GPOP-0, 41% in GPOP-Trend). The sample characteristics show a similar pattern of key variables at both survey time points (see Table 2).

**Table 2:** Sample characteristics

	GP			OHP		
	GPOP-0 (*n* = 585)	GPOP-Trend (*n* = 482)	Total (*n* = 1,058)	GPOP-0 (*n* = 473)	GPOP-Trend (*n* = 532)	Total (*n* = 1,014)
Male in %	62.0% (*n* = 360)	49.5% (*n* = 236)	56.3% (*n* = 596)	53.3% (*n* = 252)	48.0% (*n* = 253)	50.5% (*n* = 505)
Female in %	38.0% (*n* = 221)	50.3% (*n* = 240)	43.6% (*n* = 461)	46.7% (*n* = 221)	51.8% (*n* = 273)	49.4% (*n* = 494)
Divers in %	0.0% (*n* = 0)	0.2% (*n* = 1)	0.1% (*n* = 1)	0.0% (*n* = 0)	0.2% (*n* = 1)	0.1% (*n* = 1)
Age in years: Mean ± SD	54.8 ± 8.2 (*n* = 565)	55.5 ± 9.7 (*n* = 426)	55.1 ± 8.9 (*n* = 991)	52.8 ± 6.7 (*n* = 464)	55.7 ± 10.7 (*n* = 491)	54.3 ± 9.1 (*n* = 955)
Years in the profession: Mean ± SD	26.4 ± 8.8 (*n* = 575)	27.0 ± 9.9 (*n* = 467)	26.7 ± 9.3 (*n* = 1,042)	25.1 ± 7.9 (*n* = 469)	28.1 ± 11.0 (*n* = 514)	26.7 ± 9.8 (*n* = 983)
Years in medical specialty: Mean ± SD	18.3 ± 9.3 (*n* = 571)	18.3 ± 10.9 (*n* = 472)	18.3 ± 10.0 (*n* = 1,043)	17.6 ± 8.2 (*n* = 465)	19.2 ± 11.4 (*n* = 518)	18.4 ± 10.1 (*n* = 983)
Double qualification as GP or OHP in %	5.8% (*n* = 34)	1.2% (*n* = 6)	3.7% (*n* = 40)	36.8% (*n* = 174)	39.1% (*n* = 208)	38.0% (*n* = 382)
Additional qualifications in %	7.2% (*n* = 42)	5.9% (*n* = 28)	6.6% (*n* = 70)	5.2% (*n* = 25)	9.8% (*n* = 52)	7.7% (*n* = 77)
rural community (< 5.000) in %	6.0% (*n* = 35)	6.0% (*n* = 29)	6.0% (*n* = 64)	3.2% (*n* = 15)	3.4% (*n* = 18)	3.3% (*n* = 33)
small-sized community (5.000-19.999) in %	27.7% (*n* = 162)	43.2% (*n* = 208)	34.7% (*n* = 370)	14.2% (*n* = 67)	44.4% (*n* = 236)	30.1% (*n* = 303)
medium-sized community (20.000-99.999) in %	36.1% (*n* = 211)	50.8% (*n* = 245)	42.7% (*n* = 456)	34.0% (*n* = 161)	51.5% (*n* = 274)	43.3% (*n* = 435)
large-sized community (≥ 100.000) in %	30.3% (*n* = 177)	0.0% (*n* = 0)	16.6% (*n* = 177)	48.6% (*n* = 230)	0.8% (*n* = 4)	23.3% (*n* = 234)

*Abbreviations*: *GPOP-0study* survey 2014/2015, *GPOP-Trend *survey 2023/2024, *GP *general practitioner, *OHP *occupational health physician, *n *number of valid answers, *SD *standard deviation

### Experiences with cooperation during the COVID-19 pandemic

Our first research question (RQ 1) focused on how GPs and OHPs experienced their cooperation during the COVID-19 pandemic in retrospect. Almost 70% of the GPs and around 38% to 50% of the OHPs indicated not having at all been cooperating with the other professional group during the COVID-19 pandemic (for further results see Additional File 5). In cases where cooperation took place, OHPs rated the cooperation with the other professional group significantly more positively in retrospect than GPs their cooperation with OHPs. In OHPs, the two sum scores for the perceived quality of cooperation were > 8, which represents in our opinion good experiences (see Table 3). The identified statistically significant differences between the two professional groups showed for both sum scores a moderate effect according to Cohen [31].

**Table 3:** Cooperation of GPs and OHPs during the COVID-19 pandemic: experiences in retrospect (sum scores; *N* = 1014; survey time: only GPOP-Trend)

Experiences of cooperation with the other professional group when counselling an individual…	GPs	OHPs	Differences between professional groups ^d^
	Mean ± SD, Median; range; *n*_valid_		*p*; effect size
a) with increased COVID-19 risk (3 items) ^a, b, c^	5.8 ± 3.2, 5; 1–15; 163	8.3 ± 3.6, 8; 1–15; 340	*p*<.001; d=-0.33
b) suffering from Long COVID (3 items) ^a, b, c^	6.3 ± 3.6, 5; 1–15; 141	8.5 ± 3.6, 9; 1–15; 271	*p*<.001; d=-0.28

*Abbreviations:* *GP *general practitioner, *OHP *occupational health physician, *SD *standard deviation, *n*_*valid*_ number of valid answers

^a^ ‘No experience’ excluded; ^b^ sum score of five-point Likert scaled items ranging from 1 to 15 points (≥ eight points: good experiences); ^c^ Wording of items regarding counselling an individual with increased COVID-19 risk or regarding an individual suffering from Long COVID: (1) My contact with the responsible GP/OHP was helpful for my OHP/GP advice to the relevant persons, (2) My contact with the responsible GP/OHP was helpful in my decision regarding measures for these people, (3) In my opinion, the responsible GP/OHP gave the affected persons good advice; ^d^ Mann-Whitney U test, *r* < .10 = small effect/difference, *r* < .50 = medium effect/difference and *r* ≥ .50 = large effect/difference

### Attitudes towards cooperation of both professional groups – trends over time

RQ 2 focused on potential differences in the attitudes towards cooperation regarding the respective other professional group by analysing the ratings regarding the four attitudes resulting from exploratory factor analysis.

The attitude focusing on patients´ outcomes (“Benefits for patient care through the involvement of OHPs”) received highest approval by both professional groups at both survey time points (medians of GP/OHPs = 3.3/4.3; scale from 1 = agree not at all to 5 = agree definitely) with the difference between both professional groups being statistically significant (Table 4). At both time points, GPs agreed significantly more on the two rather critical attitudes towards the work of OHPs than members of the latter professional group: “He who pays the piper calls the tune” - GPs median 2.7 (GPOP-0) or 2.3 (GPOP-Trend) vs. OHPs: median 1.3 at both time points; “Poaching in foreign hunting grounds” - GPs median 2.0 (GPOP-0) or 1.5 (GPOP-Trend) vs. OHPs: median 1.5 at both time points (Table 4).

**Table 4:** Attitudes towards cooperation between both professional groups: descriptive results for both survey time points and differences between professional groups

		GP	OHP	Differences between professional groups ^b^
Manifestation of attitudes	Survey time point	Mean ± SD, median; n_valid_	Mean ± SD, median; n_valid_	*p;* effect size
“He who pays the piper calls the tune” ^a^	GPOP-0	2.7 ± 1.0, 2.7; 569	1.5 ± 0.6, 1.3; 469	*p*<.001; d_Cohen_=-0.60
	GPOP-Trend	2.4 ± 0.9, 2.3; 477	1.5 ± 0.6, 1.3; 529	*p*<.001; d_Cohen_=-0.51
	Total	2.6 ± 1.0, 2.5 1,046	1.5 ± 0.6, 1.3; 998	*p*<0.001**;** d_Cohen_**=-**0.56
“Well-meant. but not done well” ^a^	GPOP-0	2.3 ± 0.7, 2.3; 571	3.2 ± 0.7, 3.2; 469	*p*<.001; d_Cohen_=-0.54
	GPOP-Trend	2.2 ± 0.7, 2.2; 477	3.1 ± 0.7, 3.2; 529	*p*<.001; d_Cohen_=-0.54
	Total	2.3 ± 0.7, 2.2; 1,048	3.2 ± 0.7, 3.2; 998	*p*<0.001; d_Cohen_=-0.54
“Benefits for patient care through the involvement of occupational health physicians” ^a^	GPOP-0	3.2 ± 0.8, 3.3; 570	4.3 ± 0.7, 4.3; 470	*p*<.001; d_Cohen_=-0.59
	GPOP-Trend	3.3 ± 0.9, 3.3; 477	4.2 ± 0.7, 4.3; 529	*p*<.001; d_Cohen_=-0.46
	Total	3.3 ± 0.9, 3.3; 1,047	4.2 ± 0.7, 4.3; 999	*p*<0.001; d_Cohen_=-0.53
“Poaching in foreign hunting grounds” ^a^	GPOP-0	2.1 ± 1.0, 2.0; 566	1.7 ± 0.8, 1.5; 468	*p* < .001; d_Cohen_=-0.20
	GPOP-Trend	1.9 ± 0.9, 1.5; 477	1.7 ± 0.7, 1.5; 528	*p*=.007; d_Cohen_=-0.08
	Total	2.0 ± 1.0, 2.0; 1,043	1.7 ± 0.7, 1.5; 996	*p*<0.001; d_Cohen_=-0.15

*Abbreviations*: *GPOP-0 study *survey 2014/2015, *GPOP-Trend *survey 2023/2024, *GP *general practitioner, *OHP *occupational health physician, *SD *standard deviation, *n *number of valid answers

^a^attitudes resulting from exploratory factor analysis, for single items please see Table 1, Likert scale: 1 = agree not at all, 5 = agree definitely; ^b^Mann-Whitney U test, r < .10 = small effect/difference, r < .50 = medium effect/difference and r ≥ .50 = large effect/difference

OHPs indicated significantly higher approval for the critical attitude towards the work of GPs (“Well meant, but not done well” - GPs median 2.3 (GPOP-0) or 2.2 (GPOP-Trend) vs. OHPs: median 3.2 at both time points) (Table 4). Over time, there was only a modest decline in the strength of expression of attitudes in both professional groups, but an overly critical view of the GPs towards the work of OHPs (i.e. “He who pays the piper calls the tune” and “Poaching in foreign hunting grounds”) weakened as a trend (Table 4). For the latter, the effect of the difference between both professional groups was only small according to Cohen whereas it was rather large regarding the three other attitudes (Table 4).

### Influence of experiences during the COVID-19 pandemic on attitudes towards cooperation of GPs and OHPs

For the third RQ (RQ 3) we investigated how experiences during the COVID-19 pandemic and which sociodemographic and job-specific characteristics have an influence on attitudes towards cooperation of both professional groups. We therefore performed regression analyses, investigating the four attitudes (see Table 5). In every of the four regression models, professional group prevailed as strongest predictor, influencing the underlying attitudes. Other sociodemographic and job-specific predictors were in some cases gender and years in medical specialty, but with only low influence. Only with regard to the attitude focusing on patients´ outcomes (“Benefits for patient care through the involvement of OHPs”), experiences with the other professional group during the COVID-19 pandemic (i.e. sum score gathering the three items on counselling individuals suffering from Long COVID, see Table 3) remained as predictor with statistically significant effect in the parsimonious model, but with low influence compared to the predictor professional group.

**Table 5:** Influence of experiences with cooperation during the COVID-19 pandemic and sociodemographic variables on attitudes towards cooperation with the other professional group (parsimonious models, results of multivariate linear regression analyses; only GPOP-Trend data)

Attitudes	Characteristics (predictors)	B	SE(B)	β	*p*	95%CI
“He who pays the piper calls the tune” (r^2^ = 0.26)	(Constant)	2.50	0.10		0.000	2.303–2.698
	Professional group ^a, b^	-0.96	0.08	-0.51	0.000	-1.12–0.79
	Double qualification ^b^	-	-	-	-	-
	Gender ^b^	-	-	-	-	-
	Age	-	-	-	-	-
	Years in the profession	-	-	-	-	-
	Years in medical specialty ^a^	0.00	0.00	-0.01	0.785	-0.01-0.01
	Experience of cooperation with the other professional group when counselling an individual with increased COVID-19 risk ^c^	-	-	-	-	-
	Experience of cooperation with the other professional group when counselling an individual suffering from Long COVID ^d^	-	-	-	-	-
“Well-meant. but not done well” (r^2^ = 0.29)	(Constant)	2.21	0.10		0.000	2.02–2.39
	Professional group ^a, b^	0.98	0.08	0.54	0.000	0.82–1.14
	Double qualification ^b^	-	-	-	-	-
	Gender ^b^	-	-	-	-	-
	Age	-	-	-	-	-
	Years in the profession	-	-	-	-	-
	Years in medical specialty ^a^	0.00	0.00	-0.02	0.728	-0.01-0.01
	Experience of cooperation with the other professional group when counselling an individual with increased COVID-19 risk ^c^	-	-	-	-	-
	Experience of cooperation with the other professional group when counselling an individual suffering from Long COVID ^d^	-	-	-	-	-
“Benefits for patient care through the involvement of occupational health physicians” (r^2^ = 0.31)	(Constant)	2.51	0.21		0.000	2.10–2.91
	Professional group ^a, b^	0.95	0.09	0.47	0.000	0.77–1.14
	Double qualification ^b^	-	-	-	-	-
	Gender ^b^	0.23	0.09	0.12	0.009	0.06–0.40
	Age	-	-	-	-	-
	Years in the profession	-	-	-	-	-
	Years in medical specialty ^a^	0.00	0.00	-0.02	0.698	-0.01-0.01
	Experience of cooperation with the other professional group when counselling an individual with increased COVID-19 risk ^c^	-	-	-	-	-
	Experience of cooperation with the other professional group when counselling an individual suffering from Long COVID ^d^	0.05	0.01	0.19	0.000	0.03–0.07
“Poaching in foreign hunting grounds” (r^2^ = 0.04)	(Constant)	2.19	0.11		0.000	1.98–2.40
	Professional group ^a, b^	-0.31	0.09	-0.18	0.001	-0.48–-0.13
	Double qualification ^b^	-	-	-	-	-
	Gender ^b^	-	-	-	-	-
	Age	-	-	-	-	-
	Years in the profession	-	-	-	-	-
	Years in medical specialty ^a^	-0.01	0.00	-0.11	0.038	-0.02-0.00
	Experience of cooperation with the other professional group when counselling an individual with increased COVID-19 risk ^c^	-	-	-	-	-
	Experience of cooperation with the other professional group when counselling an individual suffering from Long COVID ^d^	-	-	-	-	-

*Abbreviations*: *B *regression coefficient, *β *standardized regression coefficient beta, *CI *confidence interval, *GPOP-0 *survey 2014/2015, *GPOP-Trend *survey 2023/2024, *p *significance value, *r*^*2*^ explained variance by predictor variables in the parsimonious model, *SE(B) *standard error of beta, *‘-’* not relevant in that model

^a^ Adjusted in regression model; backward elimination of other variables from the model; imputed data

^b^ Coding of non-numeric variables: profession group (0 = general practitioner, 1 = occupational health physician), double qualification as GP and OHP (0 = no, 1 = yes), gender (0 = male, 1 = female)

^c^ sum score gathering 3 items regarding counselling an individual with increased COVID-19 risk (see Table 3)

^d^ sum score gathering 3 items regarding counselling an individual suffering from Long COVID (see Table 3)

### Influence of sociodemographic and job-specific characteristics on attitudes towards cooperation of GPs and OHPs over time

The fourth RQ (RQ 4) focused on the question which sociodemographic and job-specific characteristics influenced attitudes of GPs and OHPs towards cooperation over time (2014–2024). Analyzing the possible effects of survey time point, professional group, double qualification as GP and OHP, gender, age, years in the profession, and years in medical specialty by parsimonious models revealed that survey time was significantly relevant in three models (attitudes: “He who pays the piper calls the tune”, “Well-meant. but not done well”, and “Poaching in foreign hunting grounds”), but of lesser importance than professional group which was the strongest predictor in all four regression models. Other variables were of varying importance depending on the single attitude (Table 6).

**Table 6:** Influence of survey time points, sociodemographic and job-specific characteristics on attitudes towards cooperation of GPs and OHPs (parsimonious models, results of multivariate linear regression analyses; data from GPOP-0 and GPOP-Trend)

		Total (*n* = 2,072)			
Attitudes	Predictor	B	SE_(B)_	β	p
“He who pays the piper calls the tune” (r^2^ = 0.32)	*(Constant)*	1.79	0.08		< 0.001
	Survey time point ^a, b^	-0.18	0.01	-0.09	< 0.001
	Professional group ^a, b^	-1.02	0.01	-0.53	< 0.001
	Double qualification ^b, c^	-	-	-	-
	Gender ^b^	-0.10	0.02	-0.05	< 0.001
	Age	0.03	0.00	0.28	< 0.001
	Years in the profession	-0.02	0.00	-0.21	< 0.001
	Years in medical specialty ^a^	0.00	0.00	-0.05	< 0.001
“Well-meant. but not done well” (r^2^ = 0.28)	*(Constant)*	2.34	0.02		< 0.001
	Survey time point ^a, b^	-0.08	0.01	-0.05	< 0.001
	Professional group ^a, b^	0.87	0.01	0.52	< 0.001
	Double qualification ^b, c^	0.04	0.02	0.02	0.015
	Gender ^b^	-	-	-	-
	Age	-	-	-	-
	Years in the profession	-	-	-	-
	Years in medical specialty ^a^	0.00	0.00	-0.01	0.072
“Benefits for patient care through the involvement of occupational health physicians” (r^2^ = 0.28)	*(Constant)*	3.32	0.08		< 0.001
	Survey time point ^a, b^	0.02	0.04	0.01	0.560
	Professional group ^a, b^	0.95	0.03	0.51	< 0.001
	Double qualification ^b, c^	-	-	-	-
	Gender ^b^	0.12	0.03	0.07	< 0.001
	Age	-	-	-	-
	Years in the profession	-0.01	0.00	-0.09	0.003
	Years in medical specialty ^a^	0.00	0.00	-0.01	0.795
“Poaching in foreign hunting grounds” (r^2^ = 0.05)	*(Constant)*	1.99	0.09		< 0.001
	Survey time point ^a, b^	-0.16	0.02	-0.09	< 0.001
	Professional group ^a, b^	-0.30	0.02	-0.16	< 0.001
	Double qualification ^b, c^	-	-	-	-
	Gender ^b^	-0.09	0.02	-0.05	< 0.001
	Age	0.01	0.00	0.10	< 0.001
	Years in the profession	-0.01	0.00	-0.09	< 0.001
	Years in medical specialty ^a^	-0.01	0.00	-0.06	< 0.001

*Abbreviations*: *B *regression coefficient, *SE*_*(B)*_ standard error of beta, *β *standardized regression coefficient beta, *p *significance value, *r*^*2*^ explained variance by influencing variables in the model, *‘-’ *not relevant in that model

^a^ Adjusted predictor in the multivariate linear regression model; backward elimination of other variables from the model; imputed data

^b^ Coding of non-numeric variables: Survey time (0 = GPOP-0, 1 = GPOP-Trend), professional group (0 = GP, 1 = OHP), double qualification as GP and OHP (0 = no, 1 = yes), gender (0 = male, 1 = female)

^c^ Double qualification as GP and OHP

## Discussion

The COVID-19 pandemic had a huge impact on societies all over the world, and particularly on people in working age in healthcare services [34, 35]. The pandemic also had a substantial effect on the whole working population and how they participated in their daily work. This was particularly evident among specific risk groups: For example women took more responsibility for care work [36, 37], or people with work disabilities or underlying health conditions associated with a higher risk to acquire SARS-CoV-2 infections or severe COVID-19 had a vulnerable labour market position during the COVID-19 pandemic [38, 39].

There were several opportunities and possible reasons for cooperation between GPs and OHPs during the COVID-19 pandemic. Yet, to our knowledge, so far, there have been no studies that examine this aspect relevant to healthcare provision for people in working age. We therefore conducted a trend study to describe and analyse experiences and attitudes regarding cooperation among GPs and OHPs over time, and especially before, during and after the COVID-19 pandemic.

### Interpretation of findings and comparison with previous literature

Regarding our research question RQ 1, most GPs (70%) and many OHPs (38%) stated that there was hardly any cooperation between these two groups during the COVID-19 pandemic. In our opinion, this result is surprising given the major challenges caused by the COVID-19 pandemic for healthcare personnel. Dealing with patients who suffer for example from Long / Post-COVID represents an interdisciplinary and interprofessional task as it is described e.g. in the German medical guideline focusing on care for patients with Long / Post-COVID [22, 40]. In this document, amongst others rehabilitation and work participation are addressed explicitly. A number of studies consider cooperation and communication among healthcare workers as essential for dealing with the challenges caused by the COVID-19 pandemic, and also for the management of future pandemics [41–44]. In case that the surveyed physicians indicated cooperation during the COVID-19 pandemic, the respective experience was rated more positively by OHPs than by GPs.

Considering RQ 2, GPs agreed more on the two attitudes which express some critical attitudes towards OHPs (“He who pays the piper calls the tune” and “Poaching in foreign hunting grounds”), whereas OHPs indicated higher approval for the attitude which summarizes a rather critical view towards GPs (“Well-meant, but not done well”) and the addressing “Benefits for patient care through the involvement of occupational health physicians”. Yet, the latter was rated rather positively by GPs, too.

The different rating of these attitudes towards cooperation might hinder cooperation. Barriers for interprofessional cooperation between general practice and occupational health care were also described for assistant practitioners and practice nurses in general and occupational health care [45]. The further investigation of time trends in our sample demonstrated that attitudes among GPs and OHPs did hardly changed over time, i.e. before and after the COVID-19 pandemic. We found only slight declines within the attitudes of both professional groups in the course of time, and therefore assume that pre-existing beliefs within the professional groups still exist, but have weakened somewhat over time. Stratil et al. used in their study with GPs, OHPs and rehabilitation physicians Social Identity Theory as possible explanation for attitudes and intergroup behaviour [46]. According to this theory, individuals with the same social categories (for example being an OHP or being a GP) perceive themselves as members of a social group (in-group), and this perception contributes to the social identity of this person [46, 47]. Other persons are therefore seen as members of the in or out-group, and this membership shapes the inner attitude toward the respective other group [46, 47] – and consequently the behaviour [48]. In addition to this possible explanation, in the daily work of both professional groups there are regulatory barriers to collaboration, too. These include different organizational and financial frameworks and different approaches to care that originate from the differing areas of responsibilities (for example, GPs focus on the curative care of their patients versus OHPs focus more on the preventive care of employees).

In RQ 3, we investigated the possible impact of the experiences during the COVID-19 pandemic and sociodemographic and job-specific characteristics on attitudes regarding cooperation between both professional groups. We found only in the model for the attitude “Benefits for patient care through the involvement of occupational health physicians” a small but statistically significant influence of the experiences during the COVID-19 pandemic (i.e. the cooperation in counselling a person with Long / Post COVID), whereas socialization within the respective professional group was the most important predictor in the regression models for all four attitudes which corresponds well with the Social Identity Theory mentioned above.

Focusing on a possible effect of the course of time instead of the experiences during the COVID-19 pandemic (RQ 4), survey time was relevant in three models (attitudes: “He who pays the piper calls the tune”, “Well-meant. but not done well”, and “Poaching in foreign hunting grounds”), but of lesser importance than professional group. Similar to RQ3, socialization within a profession group, i.e. as a GP or OHP, was the main predictor in the regression models for all four attitudes. Other predictors (e.g., gender, age, years of professional experience, dual qualifications, and years of professional experience in medical specialty) were of varying importance depending on the regression model for trends of the single attitudes. It appears that more critical views towards the work of occupational health physicians weakened slightly over time, particularly among men and physicians of higher age or with more years of professional experience, while views focusing on the possible benefits for patients through involvement of OHPs increased slightly over time, particularly among women and younger physicians. In a recent qualitative interview study, gender and professional experience were mentioned as relevant influencing factor regarding effective interprofessional teamwork in the operating room [49], and the authors of the study emphasis the value of teaching strategies to recognize (and counteract) unconscious biases related to e.g. gender, ethnicity, and additional social identity factors [50].

### Practical recommendations

So far, many authors and studies have focused on interventions to foster cooperation between GPs and OHPs. They have suggested to focus on more communication and information about the other professional group and their job responsibilities, possibility of in house-hospitations in the other working field, and of course more information during medical education, vocational training and continuous medical education [3, 13, 51]. Based on their empirical findings, some authors suggested concrete operational support for processes related to patient care, such as brief medical reports or the possibility of providing financial support for services to foster interdisciplinary cooperation between OHPs and GPs or rehabilitation physicians [3, 13, 52]. In some interventional studies, joint training courses and training sessions for specific consulting occasions were implemented [10, 51].

Beside these approaches and recommendations, we suggest to encourage GPs and OHPs to get to know each other better, but at the same time not to expect too much from it. GPs have to face many challenging problems within their patients (chronic illnesses, multimorbidity, demographic change [1]) and many organisational challenges characterising the demands of their jobs [53–56] which may have increased during the COVID-19 pandemic [57]. Seemingly simple issues such as sick notes or other reasons for consultations by people of working age possibly are not a priority in their everyday work. Perhaps patients who are presumably young and able to work, but who critically question their ability to work and show no relevant interest in changing their health and work behaviour, are also difficult to handle for GPs. Similarly, other frequent reasons for consultation (e.g. common mental disorders) or services that are not directly accessible through primary care (e.g., prevention services offered by the German Pension Insurance Fund) or are complex (return to work) could be used to foster cooperation between GPs and OHPs encouraging GPs to let the OHPs take the lead.

An intervention study by van Amstel et al. focusing on cooperation between GPs and OHPs revealed no significant relevant outcome for patients [51], despite it showed minor positive changes in the social medical counselling of patients by OHPs and GPs and an increase in patient satisfaction. However, the intervention did not lead to a relevant reduction in sick leave due to low back pain [51]. These results were also confirmed by another study from Faber et al. [10]. Based on the results of Stratil et al. [46] and on the Social Identity Theory [47], maybe it is more promising to define clear interfaces and design them well focusing on the concrete outcome for patients (e.g., clear responsibilities, clear treatment pathways, implementation of simple forms, accountable care [58]), including work-related topics in disease management programs (DMP) and clinical guidelines so that they are “kept in mind”. This is the case for example in the German clinical practice guideline on non-specific low back pain and DMP on chronical low back pain, which explicitly cover work-related aspects (e.g. reducing days of sick leave) and recommendations concerning return to work [59, 60]. By focusing on non-specific low back pain, one frequent reason for consulting a GP is addressed that is also relevant for work ability and employability. This approach works for example well in the Netherlands [61, 62].

A Dutch study conducted in 2006 among long-term sick listed employees due to mental health problems showed that OHPs were more involved in measures related to work than GPs [63]. In our opinion, more research is needed on what GPs think and what motivates them to ask and address work-related aspects in their consultations, from a pathogenetic as well as a salutogenetic perspective. Ehmann et al. for example investigated to what extent patients discussed their subjective future work ability with their general practitioner, and described that this only rarely happened [64]. We also need more information how OHPs can support, and maybe offload GPs in their daily work. This can be done by adjusting the mandate framework in the Social Security Codes or regulations on health care covered by the statutory health insurance. First approaches exist in the areas of vocational reintegration, especially graded return to work [65] and the treatment of Long / Post COVID patients [66]. Also, the German Prevention Act provides impulses for greater involvement of OHPs (for example through vaccinations at workplaces) [67]. During the COVID-19 pandemic, in Germany, OHPs were deeply involved in COVID-19 vaccination programs as soon as sufficient amounts of COVID-19 vaccines were available [68]. At the same time, however, certain barriers remain in place in Germany, such as special regulations governing the restricted access to the general electronic patient record by occupational health physicians [69].

A possible topic for more involvement of OHPs is return to work. Return to work is complex and requires comprehensive support [70] - something that GPs are often unable to provide and could be more supported by OHPs. However, GPs need to understand that it is the responsibility of GPs to support a successful return to work process by motivating patients accordingly. In the Netherlands, OHPs are particularly more involved in return-to-work processes [71] than in Germany. A recent literature review also demonstrates that it is worthwhile for low back pain patients when OHPs are involved more in the return-to-work process [72]. Even in cases of major depression, involving OHPs and focusing on work or return to work could be beneficial for patients [73] and proved to be effective in a randomized controlled trial in the Netherlands [74]. Poor communication and cooperation between healthcare providers can lead to different treatment advices and hinder a good coordination of care for patients [61]. A former study for example revealed among GPs, OHPs and insurance physicians’ different recommendations for sick leave for patients in five European countries, and concluded that observed differences may be originating from socialization in other working conditions, relationships with patients and trainings as well as healthcare systems [75]. Return to work after a severe course of COVID-19 or for patients suffering from Long or Post COVID is another challenging and continuing task for GPs and OHPs and benefits from a careful clinical and occupational health approach, too [22].

Overall, based on previous studies in this field, we believe that interventions targeting both professional groups should focus more on improving patient care and patients´ outcomes than solely on improving cooperation between these groups as, according to Social Identity Theory, socialization within a group remains a key driver of attitudes toward other groups and therefore difficult to influence. Therefore, problems with care provision, roles, and interfaces should be structured so clearly that cooperation is either unnecessary or arises automatically and does not depend on individual attitudes. This approach can contribute in our opinion to the Sustainable Development Goal (SDG) “Decent work” [76] of GPs and OHPs.

### Strengths and limitations

There are several strengths in our study, foremost among them being its longitudinal approach in the form of a trend study. We achieved satisfying response rates among GPs and OHPs compared with other studies in this field [9, 51]. The almost identical research methods and the same questionnaire used in GPOP-0 and GPOP-Trend study should also be highlighted as a strength, and make the different samples comparable.

Despite these strengths, we also have to mention several limitations. One limitation lies in our drawn sample or more specifically in the study participants. Probably, only highly motivated GPs and OHPs participated in our study. We also detected differences between the sample in GPOP-0 and GPOP-Trend regarding city size (number of inhabitants). Unfortunately, there were less GPs and OHPs with practices in larger cities represented in GPOP-Trend. We conducted an additional non-responder analysis using the available postal codes from the GPOP trend data and looked at the city size for GPs and OHPs that were asked to participate in the survey but did not respond. The non-responder analysis showed a similar distribution and concentration in small and medium-sized communities as among the surveyed physicians. Therefore, we can exclude any distortion in our sample with regard to the variable city size. With respect to the proportion of GPs with double qualifications, our numbers in GPOP-0 (5%) correspond to the total population of GPs in Germany (5.8%) who had an additional qualifications in occupational medicine in 2015 [77] whereas in GPOP-Trend, our numbers (1.2%) are considerably lower compared to the total population of GPs in Germany (6.5%) who were qualified in occupational medicine in 2024, too [78]. We therefore cannot assume that the participants from either group form a representative sample. The data in our trend study originated from two cross-sectional studies with questionnaires. We relied on self-reports of the study participants without the use of other research methods, such as participatory observation in the workplace or qualitative interviews. Furthermore, we addressed the cooperation within the COVID-19 pandemic only in retrospect with two sum scores each consisting of three single items. Hence, the assessment of cooperation during the COVID-19 pandemic is somewhat limited. It is also possible that respondents had different understandings of Long COVID as reason for consultation, as we did not explain our definition in the questionnaire, nor did we explain the difference between Long COVID and Post-COVID in the information sheets, for example.

## Conclusion

In our trend study, we identified little cooperation among GPs and OHPs in terms of the challenges arising from the COVID-19 pandemic, and could describe that attitudes towards cooperation with the respective other professional group persist over time. Cooperation is still an important issue for these two groups, but in our opinion thinking about fostering cooperation should be reconsidered. We suggest interventions targeting primarily on patient outcomes and the design of clear interfaces, for example in complex topics like return-to-work processes and the management of Long / Post COVID patients, and less on sole improvements of cooperation between GPs and OHPs. This approach may be more promising for patients´ benefit and thus - in a second step - for the physicians, too.

## Supplementary Information

Additional File 1. Items regarding experiences of cooperation with the other professional group when counselling an individual with increased COVID-19 risk.

Additional File 2. Attitudes towards common and separate working fields and responsibilities (factor loading from exploratory factor analysis; 13-item solution out of 17 items).

Additional File 3. Psychometric properties for “Agreement to statements concerning working fields and tasks”, 13-item solution out of 17 items (imputed data; *N*= 2072).

Additional File 4. Floor and ceiling effects of factors “Agreement to statements concerning working fields and tasks”; raw data; *N*=2,072.

Additional File 5. Experiences of collaboration with the other professional group when counselling an individual with COVID-19 or Long COVID: Percentage of physicians who have not experienced the situation.

## Data Availability

We support the free availability of research data and the FAIR principles. However, due to the same methodological approach chosen, the participants in the trend study were not informed about the storage of research data in a repository. For this reason, the datasets generated and/or analysed during the current study are not publicly available, but are available from the corresponding author on reasonable request.

## Abbreviations

BW: Baden-Württemberg
COVID-19: coronavirus disease 2019
DMP: disease management program
GDPR: general data protection regulation of the European Union
GPs: general practitioners
NRW: North Rhine-Westphalia
OHPs: occupational health physicians
OR: odds ratio
P: significance value
RQ: research questions
SARS-CoV-2: severe acute respiratory syndrome-coronavirus-2
SDG: Sustainable Development Goal
