# Supplementary material for: Even the COVID-19 pandemic didn´t change anything: insights from a trend study on the cooperation of general practitioners and occupational health physicians in Germany
Source: BMC Prim Care. 2026 Jul 9;27:269. doi: 10.1186/s12875-026-03463-7 (PMC13374197; doi:10.1186/s12875-026-03463-7)
Supplement: Supplementary file 1 — Additional File 1. Items regarding experiences of cooperation with the other professional group when counselling an individual with increased COVID-19 risk. [file 12875_2026_3463_MOESM1_ESM.docx]

## Additional File 1

**Items regarding experiences of cooperation with the other professional group when counselling an individual with increased COVID-19 risk**

During the COVID-19 pandemic, people at increased risk of SARS-CoV-2 infection or severe COVID-19 disease were given medical advice. What experiences have you had as a GP/OHP in collaborating with OHPs/GPs?

Im Rahmen der COVID-19-Pandemie wurden Personen mit einem erhöhten Risiko für eine SARS-CoV-2-Infektion oder einen schweren COVID-19-Verlauf ärztlich beraten. Welche Erfahrungen haben Sie als Hausarzt/Betriebsarzt hierbei in der Zusammenarbeit mit Betriebsärzten/Hausärzten gemacht?

| **Variable** | **Items in English /German** | **Scale** |
| --- | --- | --- |
| **Experienced helpfulness of the interdisciplinary cooperation** | My contact with the responsible GP/OHP was helpful for my OHP/GP advice to the relevant persons.  Mein Kontakt mit dem zuständigen Hausarzt/Betriebsarzt war hilfreich für meine betriebsärztliche/ hausärztliche Beratung der entsprechenden Personen. | 5-point Likert scale (1=do not agree, 5=fully agree; 0=situation not experienced) |
| **Decisions for measures and perception** | My contact with the responsible GP/OHP was helpful in my decision regarding measures for these people.  Mein Kontakt mit dem zuständigen Hausarzt/Betriebsarzt war hilfreich für meine Entscheidung hinsichtlich Maßnahmen für diese Personen. |  |
| **Rating of the counselling of the respective other profession** | In my opinion, the responsible GP/OHP gave the affected persons good advice.  Meiner Einschätzung nach hat der zuständige Hausarzt/Betriebsarzt die betroffenen Personen gut beraten. |  |

**Items regarding experiences of cooperation with the other professional group when counselling an individual suffering from Long COVID**

What experiences have you had as a GP/OHP in working with OHPs/GPs to support employees with Long COVID?

Welche Erfahrungen haben Sie als Hausarzt/Betriebsarzt in der Zusammenarbeit mit Betriebsärzten/Hausärzten gemacht bei der Begleitung von Beschäftigten mit Long COVID?

| **Variable** | **Items in English / German** | **Scale** |
| --- | --- | --- |
| **Experienced helpfulness of the interdisciplinary cooperation** | My contact with the responsible GP/OHP was helpful for my OHP/GP advice to the relevant persons.  Mein Kontakt mit dem zuständigen Hausarzt/Betriebsarzt war hilfreich für meine betriebsärztliche/ hausärztliche Beratung der entsprechenden Personen. | 5-point Likert scale (1=do not agree, 5=fully agree; 0=situation not experienced) |
| **Decisions for measures and perception** | My contact with the responsible GP/OHP was helpful in my decision regarding measures for these people.  Mein Kontakt mit dem zuständigen Hausarzt/Betriebsarzt war hilfreich für meine Entscheidung hinsichtlich Maßnahmen für diese Personen. |  |
| **Rating of the counselling of the respective other profession** | In my opinion, the responsible GP/OHP gave the affected persons good advice.  Meiner Einschätzung nach hat der zuständige Hausarzt/Betriebsarzt die betroffenen Personen gut beraten. |  |
