# Supplementary material for: Even the COVID-19 pandemic didn´t change anything: insights from a trend study on the cooperation of general practitioners and occupational health physicians in Germany
Source: BMC Prim Care. 2026 Jul 9;27:269. doi: 10.1186/s12875-026-03463-7 (PMC13374197; doi:10.1186/s12875-026-03463-7)
Supplement: Supplementary file 2 — Additional File 2. Attitudes towards common and separate working fields and responsibilities (factor loading from exploratory factor analysis; 13-item solution out of 17 items). [file 12875_2026_3463_MOESM2_ESM.docx]

## Additional File 2

**Attitudes towards common and separate working fields and responsibilities (factor loading from exploratory factor analysis; 13-item solution out of 17 items)**

|  |  | **GP** | | | | | | | | | | | | **OHP** | | | | | | | | | | | |
| --- | --- | --- | --- | --- | --- | --- | --- | --- | --- | --- | --- | --- | --- | --- | --- | --- | --- | --- | --- | --- | --- | --- | --- | --- | --- |
|  | **Survey time point** | **GPOP-0**  **(n= 585)** | | | | **GPOP-Trend**  **(n= 482)** | | | | **Total**  **(n=1,067)** | | | | **GPOP-0**  **(n=473)** | | | | **GPOP-Trend**  **(n= 532)** | | | | **Total**  **(n=1,005)** | | | |
| **Dimension** | **Items** | **D1** | **D2** | **D3** | **D4** | **D1** | **D2** | **D3** | **D4** | **D1** | **D2** | **D3** | **D4** | **D1** | **D2** | **D3** | **D4** | **D1** | **D2** | **D3** | **D4** | **D1** | **D2** | **D3** | **D4** |
| “He who pays the piper calls the tune”  (Factor 1) | OHPs' care focuses more likely on employer's well-being than on worker's well-being | **0.67** |  | 0.10 | -0.20 | **0.66** | 0.14 | -0.12 |  | **0.67** | 0.12 |  | -0.12 | 0.28 | **0.30** | **-0.39** | 0.17 | 0.26 | **0.38** | -0.23 |  | 0.28 | **0.32** | -0.31 | 0.14 |
|  | There is high risk that OHPs do not adhere to medical confidentiality towards employers | **0.79** | 0.20 |  |  | **0.81** |  |  | 0.18 | **0.80** |  | 0.18 |  |  | **0.84** |  | 0.14 |  | **0.84** |  |  |  | **0.85** |  |  |
|  | In regard to addictive disorders, there is high risk that OHPs inform employers without employees' permission | **0.76** | 0.18 |  | 0.12 | **0.81** |  |  | 0.17 | **0.79** |  | 0.15 | 0.11 |  | **0.84** | -0.10 |  |  | **0.78** |  | 0.16 |  | **0.80** |  | 0.13 |
| “Well meant, but not done well”  (Factor 2) | GPs tend to protect their patients from work | 0.21 | **-**0.15 | **0.72** | -0.20 | 0.31 | **0,62** |  | -0.10 | 0.26 | **0.67** | -0.16 |  | **0.74** |  |  |  | **0.72** | 0.14 |  |  | **0.73** |  |  |  |
|  | GPs interfere in OHPs' field of responsibility | -0.14 | **0.48** | **0.42** |  |  | **0.67** |  | 0.16 |  | **0.61** | 0.29 |  | **0.63** | 0.12 |  |  | **0.57** | 0.13 |  | 0.24 | **0.59** | 0.10 |  | 0.19 |
|  | GPs often lack knowledge of employee's work necessary for certifying sick-leave | 0.11 | 0.13 | **0.47** | 0.39 | 0.17 | **0.41** | **0.48** |  | 0.13 | **0.44** |  | **0.44** | **0.62** | -0.21 | 0.24 |  | **0.69** | -0-15 | 0.25 |  | **0.66** | -0.18 | 0.25 |  |
|  | GPs often do not consider employers' needs when certifying extension of sick-leaves |  |  | **0.59** | 0.15 | 0.25 | **0.54** | 0.29 |  | 0.13 | **0.58** |  | 0.22 | **0.64** |  | 0.10 | 0.11 | **0.64** |  |  |  | **0.64** |  |  |  |
|  | Workplace-related medical certificates often do more harm than benefit |  | 0.23 | **0.61** | 0.13 |  | **0.69** |  | 0.15 |  | **0.62** | 0.21 | 0.12 | **0.58** | 0.14 |  |  | **0.59** |  | 0.17 | -0.17 | **0.59** |  | 0.12 | -0.14 |
| “Benefits for patient care through the involvement of OHPs”  (Factor 3) | OHPs should be involved in stepwise reintegration into work | 0.17 | -0.23 | 0.14 | **0.68** |  |  | **0.76** |  |  |  | -0.12 | **0.75** |  |  | **0.77** |  |  |  | **0.73** |  |  |  | **0.76** |  |
|  | Close cooperation between GPs and OHPs can shorten times of work disability |  |  |  | **0.74** |  |  | **0.83** |  | -0.14 |  |  | **0.80** |  |  | **0.77** |  |  | -0.11 | **0.80** |  |  |  | **0.78** |  |
|  | OHPs should get remuneration for preventive services from the statutory health insurance | -0.37 | 0.18 |  | **0.44** | -0.20 | 0.26 | **0.37** | -0.24 | **-0.34** | 0.28 | -0.12 | 0.27 | 0.31 |  | **0.33** |  | 0.15 |  | **0.43** | 0.27 | 0.20 | -0.14 | **0.39** | 0.19 |
| “Poaching in foreign hunting grounds”  (Factor 4) | OHPs perform too many services that belong to the field of responsibility of GPs | 0.26 | **0.78** | 0.14 |  |  |  |  | **0.84** | 0.17 | 0.11 | **0.84** |  | 0.16 |  |  | **0.83** |  | 0.15 | -0.12 | **0.77** | 0.11 | 0.11 |  | **0.78** |
|  | OHPs interfere in GPs' field of responsibility | 0.27 | **0.80** |  | -0.11 | 0.24 | 0.13 |  | **0.79** | 0.24 |  | **0.82** |  |  | 0.18 |  | **0.80** |  |  |  | **0.81** |  |  |  | **0.82** |
|  | **Explained total variance** | 53.9% | | | | 53.0% | | | | 60.6% | | | | 55.7% | | | | 51.7% | | | | 59.1% | | | |

Abbreviations: GP=general practitioner, OHP=occupational health physician, GPOP-0=survey 2014/2015, GPOP-Trend=survey 2023/2024

Item excluded from analysis due to low factor loading: (4) GPs see OHPs as competition; (7) GPs feel criticized by OHPs when OHPs communicate remarkable medical findings to GPs, (6) GPs find OHPs' work helpful, (8) OHPs find GPs' work helpful.
