# Supplementary material for: Even the COVID-19 pandemic didn´t change anything: insights from a trend study on the cooperation of general practitioners and occupational health physicians in Germany
Source: BMC Prim Care. 2026 Jul 9;27:269. doi: 10.1186/s12875-026-03463-7 (PMC13374197; doi:10.1186/s12875-026-03463-7)
Supplement: Supplementary file 3 — Additional File 3. Psychometric properties for “Agreement to statements concerning working fields and tasks”, 13-item solution out of 17 items (imputed data; N= 2072). [file 12875_2026_3463_MOESM3_ESM.docx]

## Additional File 3

**Psychometric properties for “Agreement to statements concerning working fields and tasks”, 13-item solution out of 17 items (imputed data; N= 2072)**

|  | **Corrected item-to-total correlation (CITC) / Cronbach’s alpha** | **GP** | | | **OHP** | | |
| --- | --- | --- | --- | --- | --- | --- | --- |
| **Factors** | **Survey time point**  **Items** | **GPOP-0**  **(n= 585)** | **GPOP-Trend**  **(n= 482)** | **Total (n=1,063)** | **GPOP-0**  **(n=473)** | **GPOP-Trend**  **(n= 532)** | **Total (n=1,005)** |
| “He who pays the piper calls the tune” (factor 1) | OHPs' care focuses more likely on employer's well-being than on worker's well-being | 0.40 | 0.40 | 0.41 | *0.24* | *0.17* | *0.21* |
|  | There is high risk that OHPs do not adhere to medical confidentiality towards employers | 0.61 | 0.58 | 0.60 | 0.43 | 0.41 | 0.42 |
|  | In regard to addictive disorders, there is high risk that OHPs inform employers without employees' permission | 0.53 | 0.52 | 0.53 | 0.54 | 0.38 | 0.46 |
| “Well-meant, but not done well” (factor 2) | GPs tend to protect their patients from work | 0.30 | 0.39 | 0.34 | 0.51 | 0.49 | 0.50 |
|  | GPs interfere in OHPs' field of responsibility | *0.29* | 0.35 | 0.32 | 0.40 | 0.37 | 0.38 |
|  | GPs often do not consider employers' needs when certifying extension of sick-leaves | 0.31 | 0.40 | 0.35 | 0.42 | 0.51 | 0.47 |
|  | GPs often do not consider employers' needs when certifying extension of sick-leaves | 0.39 | 0.48 | 0.43 | 0.42 | 0.42 | 0.42 |
|  | Workplace-related medical certificates often do more harm than benefit | 0.38 | 0.45 | 0.41 | 0.35 | 0.39 | 0.37 |
| “Benefits for patient care through the involvement of occupational health physicians” (factor 3) | OHPs should be involved in stepwise reintegration into work | *0.28* | *0.28* | 0.32 | *0.23* | *0.23* | *0.23* |
|  | Close cooperation between GPs and OHPs can shorten times of work disability | 0.32 | 0.32 | 0.37 | 0.35 | 0.35 | 0.36 |
|  | OHPs should get remuneration for preventive services from the statutory health insurance | *0.14* | *0.14* | *0.19* | *0.21* | *0.21* | *0.19* |
| “Poaching in foreign hunting grounds” (factor 4) | OHPs perform too many services that belong to the field of responsibility of GPs | 0.65 | 0.48 | 0.59 | 0.40 | 0.40 | 0.40 |
|  | OHPs interfere in GPs' field of responsibility | 0.65 | 0.48 | 0.59 | 0.40 | 0.40 | 0.40 |
|  | **Cronbachs’s alpha (Factor 1 to 4)** | *F1: 0.69*  *F2: 0.57*  *F3: 0.41*  F4: 0.79 | *F1: 0.68*  *F2: 0.65*  *F3: 0.41*  *F4: 0.65* | F1: 0.70  *F2: 0.61*  *F3: 0.46*  F4: 0.74 | *F1: 0.58*  *F2: 0.67*  *F3: 0.41*  *F4: 0.57* | *F1: 0.50*  *F2: 0.68*  *F3: 0.40*  *F4: 0.57* | *F1: 0.54*  *F2: 0.68*  *F3: 0.40*  *F4: 0.57* |

Abbreviations: GP=general practitioner, OHP=occupational health physician, GPOP-0=survey 2014/2015, GPOP-Trend=survey 2023/2024. Italic letters: Low item-to-total correlation (< 0.3) and low Cronbach’s alpha (< 0.7)

Item excluded from analysis due to low factor loading: (4) GPs see OHPs as competition; (7) GPs feel criticized by OHPs when OHPs communicate remarkable medical findings to GPs, (6) GPs find OHPs' work helpful, (8) OHPs find GPs' work helpful.
