# Supplementary material for: Even the COVID-19 pandemic didn´t change anything: insights from a trend study on the cooperation of general practitioners and occupational health physicians in Germany
Source: BMC Prim Care. 2026 Jul 9;27:269. doi: 10.1186/s12875-026-03463-7 (PMC13374197; doi:10.1186/s12875-026-03463-7)
Supplement: Supplementary file 4 — Additional File 4. Floor and ceiling effects of factors “Agreement to statements concerning working fields and tasks”; raw data; N=2,072. [file 12875_2026_3463_MOESM4_ESM.docx]

## Additional File 4

**Floor and ceiling effects of factors “Agreement to statements concerning working fields and tasks”; raw data; N=2,072**

|  |  | **GP % (n _valid_)** | | | **OHP % (n _valid_)** | | |
| --- | --- | --- | --- | --- | --- | --- | --- |
| **Factors**  **(n _valid_ GPOP-0;-Trend; Total GPs; OHPs)** | **Score value** | **GPOP-0** | **GPOP-Trend** | **Total** | **GPOP-0** | **GPOP-Trend** | **Total** |
| “He who pays the piper calls the tune” (factor 1)  n(GPs) = 569; 477; 1,046  n(OHPs) = 469; 529; 998 | lowest (1) ^a^ | 6.2 (35) | 12.6 (60) | 9.1 (95) | 39.4 (185) | 40.6 (215) | 40.1 (400) |
|  | highest (5) ^b^ | 2.1 (12) | 1.5 (7) | 1.8 (19) | 0.2 (1) | 0.2 (1) | 0.2 (2) |
| “Well-meant, but not done well” (factor 2)  n(GPs) = 571; 477; 1,048  n(OHPs) = 469; 529; 998 | lowest (1) ^a^ | 4.4 (25) | 7.8 (37) | 5.9 (62) | 0.2 (1) | 0.6 (3) | 0.4 (4) |
|  | highest (5) ^b^ | 0.2 (1) | 0.0 (0) | 0.1 (1) | 1.1 (5) | 0.6 (3) | 0.8 (8) |
| “Benefits for patient care through the involvement of occupational health physicians” (factor 3)  n(GPs) = 570; 477; 1,047  n(OHPs) = 470; 529; 999 | lowest (1) ^a^ | 1.1 (6) | 3.4 (16) | 2.1 (22) | 0.0 (0) | 0.2 (1) | 0.1 (1) |
|  | highest (5) ^b^ | 4.2 (24) | 7.3 (35) | 5.6 (59) | 26.0 (122) | 21.2 (112) | 23.4 (234) |
| “Poaching in foreign hunting grounds” (factor 4)  n(GPs) = 566; 477; 1,043  n(OHPs) = 468; 528; 996 | lowest (1) ^a^ | 24.7 (140) | 32.1 (153) | 28.1 (293) | 35.9 (168) | 35.2 (186) | 35.5 (354) |
|  | highest (5) ^b^ | 2.8 (16) | 1.7 (8) | 2.3 (24) | 0.2 (1) | 0.6 (3) | 0.4 (4) |

Abbreviations: GP=general practitioner, OHP=occupational health physician, GPOP-0=survey 2014/2015, GPOP-Trend=survey 2023/2024, ^a^ “don´t agree at all”, ^b^ “agree definitely”, yellow mark : floor effect=>15% “do not agree at all” and ceiling effect=>15% “fully agree”
