# Supplementary material for: Even the COVID-19 pandemic didn´t change anything: insights from a trend study on the cooperation of general practitioners and occupational health physicians in Germany
Source: BMC Prim Care. 2026 Jul 9;27:269. doi: 10.1186/s12875-026-03463-7 (PMC13374197; doi:10.1186/s12875-026-03463-7)
Supplement: Supplementary file 5 — Additional File 5. Experiences of collaboration with the other professional group when counselling an individual with COVID-19 or Long COVID: Percentage of physicians who have not experienced the situation. [file 12875_2026_3463_MOESM5_ESM.docx]

## Additional File 5

**Experiences of collaboration with the other professional group when counselling an individual with COVID-19 or Long COVID: Percentage of physicians who have not experienced the situation**

|  | **Experiences of collaboration with the other professional group when counselling an individual with…** | | | |
| --- | --- | --- | --- | --- |
| **Items** | **…increased COVID-19 risk - situation not experienced** | | **…Long COVID - situation not experienced** | |
|  | **GP** | **OHP** | **GP** | **OHP** |
| My contact with the responsible GP/OHP was helpful for my OHP/GP advice to the relevant persons. | 68.4%  (n=318) | 46.2%  (n=242) | 72.1%  (n=336) | 57.7%  (n=297) |
| My contact with the responsible GP/OHP was helpful in my decision regarding measures for these people. | 69.9%  (n=325) | 46.0%  (n=241) | 72.4%  (n=338) | 58.1%  (n=302) |
| In my opinion, the responsible GP/OHP gave the affected persons good advice. | 68.5%  (n=319) | 38.8%  (n=203) | 70.8%  (n=329) | 48.7%  (n=252) |

Abbreviations: GP=general practitioner, OHP=occupational health physician
